# An *In Vitro* Model to Evaluate the Impact of the Soluble Factors from the Colonic Mucosa of Collagenous Colitis Patients on T Cells: Enhanced Production of IL-17A and IL-10 from Peripheral CD4^+^ T Cells

**DOI:** 10.1155/2014/879843

**Published:** 2014-09-21

**Authors:** Ashok Kumar Kumawat, Nils Nyhlin, Anna Wickbom, Curt Tysk, Johan Bohr, Olof Hultgren, Elisabeth Hultgren Hörnquist

**Affiliations:** ^1^School of Health and Medical Sciences, Örebro University, 70182 Örebro, Sweden; ^2^Department of Medicine, Division of Gastroenterology, Örebro University Hospital, 70185 Örebro, Sweden; ^3^Department of Microbiology and Immunology, Örebro University Hospital, 70185 Örebro, Sweden

## Abstract

Soluble factors from intestinal mucosal cells contribute to immune homeostasis in the gut. We have established an *in vitro* model to investigate the regulatory role of soluble factors from inflamed intestinal mucosa of collagenous colitis (CC) patients in the differentiation of T cells. Peripheral blood CD4^+^ T cells from healthy donors were polyclonally activated in the presence of conditioned medium (CM) generated from denuded biopsies (DNB) or isolated lamina propria mononuclear cells (LPMCs) from mucosal biopsies from CC patients compared to noninflamed controls, to determine proliferation and secretion of cytokines involved in T-cell differentiation. Compared to controls, we observed significantly increased production of the proinflammatory cytokines IFN-*γ*, IL-17A, IL-6, and IL-1*β* and the anti-inflammatory cytokines IL-4 and IL-10 in the presence of CC-DNB-CM. The most pronounced effect of CC-LPMC-CM on peripheral CD4^+^ T cells was a trend towards increased production of IL-17A and IL-10. A trend towards reduced inhibition of T-cell proliferation was noted in the presence of CC-DNB-CM. In conclusion, our *in vitro* model reveals implications of soluble factors from CC colonic mucosa on peripheral T cells, enhancing their production of both pro- and anti-inflammatory cytokines.

## 1. Introduction

The human gastrointestinal mucosa constitutes the largest mucosal surface area in the human body interfacing the external environment. A network of complementary regulatory interactions between different types of immune and nonimmune cells maintains mucosal homeostasis in the gut. These regulatory interactions occur in the midst of a complex mixture of proteins, known as extracellular matrix (ECM) or stroma [1, 2], which together with soluble mediators such as cytokines and growth factors from mesenchymal cells, immune cells, and epithelial cells regulate cell activation and differentiation [2]. Mesenchymal cells are actively involved in the inflammatory process in the gut and can perpetuate chronic gut inflammatory conditions like inflammatory bowel disease (IBD) [3–5]. Transforming growth factor- (TGF-) *β* is one of the potential soluble mediators in homeostatic mechanisms in the gut that downregulates effector T-cell responses in the mucosa by reducing proliferation and interferon-*γ* (IFN-*γ*) production [4, 6]. However, TGF-*β* together with IL-6 and IL-1*β* from the inflamed mucosa induces proinflammatory Th17 cells, suggesting an innate regulatory function of the gut mucosal microenvironment [4, 7, 8].

Research is ongoing to elucidate the role of soluble factors and ECM in immunopathological mechanisms in IBD [4], but no such studies have so far been performed on microscopic colitis (MC) where the inflammation is subtler. MC comprises two entities, collagenous colitis (CC) and lymphocytic colitis (LC). Both conditions are characterized by chronic nonbloody, watery diarrhoea, often associated with abdominal pain and weight loss [9–11]. The colonic mucosa is macroscopically normal or almost normal and the diagnosis relies on microscopic assessment of mucosal biopsies. CC is presented with increased densities of lymphocytes and a thickened subepithelial collagen band (≥10 *μ*m) adjacent to the basal membrane. The pathophysiological data of CC are still limited, but it is postulated to be at least partially caused by disturbed immune responses to various luminal antigen(s), such as drugs, gluten, or infectious agents, in predisposed individuals [9]. Nonsteroidal anti-inflammatory drugs (NSAIDs), proton pump inhibitors, aspirin, and selective serotonin reuptake inhibitors have been associated with CC [12]. In the majority of patients, however, no precipitating factor is found. Dysregulated myofibroblast function has also been implicated for collagen deposition in CC patients [10, 13]. Recently, we reported on increased local activation of both CD4^+^ and CD8^+^ T cells in the lamina propria and epithelium of CC patients, demonstrated as increased expression of CD45RO and the proliferation marker Ki67, using flow cytometric analysis of freshly isolated lymphocytes from colonic biopsies [14]. In addition, mucosal transcript levels of IFN-*γ*, IL-12, IL-1*β*, IL-6, IL-17A, IL-21, IL-22, and IL-23 are enhanced in the inflamed mucosa of CC patients compared to normal mucosa, together with elevated protein levels of IL-6, IL-21, and TNF [15].

Although the above findings suggest that the mucosal microenvironment is involved in CC immunopathology, the interplay between these factors and the proinflammatory activity of local mucosal T cells in CC patients has not been elucidated. We therefore investigated the role of soluble factors from the intestinal mucosa of CC patients in the regulation of T cells using a novel* in vitro* model system with the aim of mimicking the* in vivo* exposure of newly recruited peripheral blood T cells to the soluble factors in the colonic milieu of inflamed CC and normal mucosa.

## 2. Material and Methods

### 2.1. Patients

CC diagnosis was confirmed by clinical symptoms: ≥3 loose stools/day and/or abdominal pain and a macroscopically normal colonic mucosa with characteristic histopathological findings: increased numbers of lymphocytes in the epithelium and lamina propria with deposition of a ≥10 *μ*m thick subepithelial collagen layer [9]. Inclusion criteria were patients previously diagnosed with CC with clinically and histopathologically active disease. Patients with enteric infection, ischemic colitis, colonic cancer, or a previous history of Crohn's disease or ulcerative colitis were excluded. None of the patients were treated with immunosuppressive drugs or antibiotics.

We investigated colonic biopsies from 7 CC patients (female; *n* = 6) and 20 noninflamed controls (female; *n* = 11) without diarrhoeal symptoms, recruited among patients undergoing colonoscopy for examination of gastrointestinal bleeding or of abnormal radiological findings. All controls had a normal colonoscopy and histology.

Twelve biopsies from the hepatic flexure from each individual were obtained using standard biopsy forceps, placed in phosphate buffered saline (PBS), and processed within 1 hour. The colonoscopies were performed at the Division of Gastroenterology, Örebro University Hospital, Sweden, between November 2012 and November 2013.

Peripheral blood from healthy donors (*n* = 6) was collected in heparin tubes for CD4^+^ lymphocyte isolation as described below.

The study was approved by the Regional Ethical Committee of Örebro-Uppsala County, Sweden (ID no. 2008/278; 081015). All patients in this study had provided written informed consent.

### 2.2. Preparation of Conditioned Medium from the Colonic Mucosa

We investigated the influence of two different preparations of the mucosa: one where the epithelium and intraepithelial cells were removed enzymatically, the denuded biopsies (DNB), and one where collagenase was used to digest the lamina propria after the removal of the epithelium. The DNB and the isolated mononuclear cells were cultured overnight, and the latter were termed lamina propria mononuclear cells (LPMCs). The DNB fraction contains collagen and mesenchymal cells, fibroblasts, and leukocytes. The cell populations are intact in the DNB fraction, whereas the lamina propria mononuclear cells (LPMCs) fraction is composed of a free leukocyte population as well as tissue, collagen, and cell debris.

Twelve biopsies were thoroughly washed with PBS and incubated with prewarmed Hank's balanced salt solution (HBSS) (Sigma Aldrich, St. Louis, MO, USA) containing 1 mM EDTA, 20 mM HEPES, and 5% heat inactivated fetal bovine serum (FBS) at 37°C, with constant stirring 4 times (15 min), to remove the epithelial layer. Six denuded biopsies were kept in serum-free RPMI-1640 containing 20 mM HEPES, 100 *μ*g/mL streptomycin, 10 *μ*g/mL gentamycin, and 100 U/mL penicillin (hereafter referred to as culture medium) on ice until further use, and the remaining six biopsies were further digested with collagenase type VIII and DNAse I type IV (Sigma Aldrich) for 1–1.5 hrs to digest the collagen. Isolated lamina propria mononuclear cells were washed twice in PBS and resuspended in culture medium. DNBs and LPMCs were cultured in culture medium overnight at 37°C under 5% CO_2_-95% air, to generate conditioned medium (CM). CM from the DNB and LPMC fractions from CC patients and noninflamed controls, respectively, were pooled. Endotoxin levels were quantified using the Pierce LAL Chromogenic Endotoxin Quantitation Kit (Pierce, Rockford, IL, USA) according to the manufacturer's protocol. The maximum endotoxin level for DNB-CM and LPMC-CM was 4 EU/mL and 4.2 EU/mL, respectively. Total protein concentrations were determined using the Bio-Rad DC protein assay kit (Bio-Rad, Hercules, CA, USA). DNB-CM was used at the total protein concentrations of 250 and 62.5 *μ*g/mL, whereas LPMC-CM was used at the total protein concentrations 125 and 62.5 *μ*g/mL, the highest possible concentrations of the respective conditioned media. CM from the intestinal mucosa of noninflamed controls is referred to as Ctrl-DNB-CM and Ctrl-LPMC-CM, whereas CM derived from CC patients is referred to as CC-DNB-CM and CC-LPMC-CM.

### 2.3. T-Cell Proliferation and Cytokine Release Assays

CD4^+^ peripheral blood lymphocytes were isolated from healthy donors using a Human CD4^+^ T-cell Enrichment Cocktail kit (STEMCELL Technologies, Grenoble, France) according to the manufacturer's protocol. Cell viability was ~95%, as determined by Trypan blue exclusion. The purity of CD4^+^ T cells was 90–95% as determined by flow cytometric analysis (Epics Altra, Beckman Coulter, Fullerton, CA, USA).

Purified CD4^+^ PBLs were cultured in 96-well flat bottom assay plates (Sarstedt, Newton, NC, USA) precoated with 50 *μ*L of 5 *μ*g/mL anti-CD3 (UCHT1, BD Biosciences, San Diego, CA, USA) for 2 hrs at 37°C, followed by two washes with PBS.

1 × 10^5^ CD4^+^ T cells were added to the washed wells together with 1 *μ*g/mL soluble anti-CD28 (BD Biosciences) and were incubated in culture medium with the addition of 2 mM L-glutamine and 5% AB serum, with or without DNB-CM or LPMC-CM from noninflamed controls or CC patients in a total volume of 200 *μ*L. As controls, Ctrl/CC-DNB-CM and Ctrl/CC-LPMC-CM were cultured without CD4^+^ T cells, and CD4^+^ T cells alone were incubated without anti-CD3/anti-CD28. In addition, control wells containing culture medium only were included. The assay was performed in duplicate wells for cytokine analysis and triplicate wells for the proliferation assay. The cells were cultured at 37°C under 5% CO_2_-95% air for three days; thereafter, supernatants were harvested and stored at −80°C until determination of cytokine content, and T-cell proliferation was measured using the CellTiter 96 AQueous One Solution Cell Proliferation Assay (3-(4,5-dimethylthiazol-2-yl)-5-(3-carboxymethoxyphenyl)-2-(4-sulfophenyl)-2H-tetrazolium, Promega, Madison, WI, USA). Forty microliters of AQueous One Solution Reagent was added into each well and incubated at 37°C for 4 hrs in a humidified, 5% CO_2_ incubator followed by recording of the absorbance at 490 nm.

### 2.4. Cytokine Analysis

The pooled conditioned medium from inflamed CC mucosa and controls as well as supernatants from peripheral CD4^+^ T cells were analyzed for IL-1*β*, IL-4, IL-6, IL-10, IL-17A, IFN-*γ*, and TNF using the xMAP technology developed by Luminex  (Austin, TX, USA), using two Milliplex Map Kits (Cat. number HCYTOMAG-60K and Cat. number HCYP2MAG-62K), according to the manufacturer's instructions (Millipore, MA, USA). The assays were performed in duplicate and the levels of different cytokines were expressed as pg/mL, according to a standard curve with known amounts of each analyte (Millipore).

TGF-*β* levels were determined by ELISA, according to the manufacturer's instructions (BD Biosciences, San Diego, CA, USA).

Cytokine amounts were calculated as follows: cytokine amounts released by peripheral T cells incubated with CM minus cytokine amounts in CM alone. For each blood donor, the cytokine amounts released by CD4^+^ T cells incubated with CM from CC patients were compared with the cytokine amounts released by CD4^+^ T cells incubated with CM from controls.

### 2.5. Statistical Analysis

As the data obtained were not normally distributed, Wilcoxon's signed rank nonparametrical test was used for statistical comparison between groups. Differences were considered statistically significant when *P* ≤ 0.05.

## 3. Results

### 3.1. An* In Vitro* Model to Study the Impact of the Mucosal Milieu on Peripheral T Cells: Increased Production of Both Pro- and Anti-Inflammatory Cytokines by Polyclonally Activated CD4^+^ T Lymphocytes in the Presence of Soluble Factors from CC Mucosa

**Figure 1:**
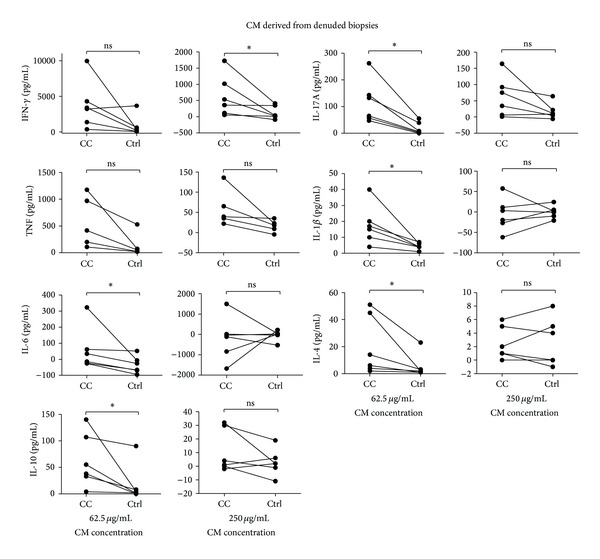
Luminex analysis for IL-1*β*, IL-4, IL-6, IL-10, IL-17A, IFN-*γ*, and TNF production by anti-CD3 plus anti-CD28 stimulated CD4^+^ peripheral blood T cells (*n* = 6; for TNF analysis, *n* = 5) cultured for 3 days in the presence of two concentrations of CM derived from denuded biopsies (DNB) from inflamed CC mucosa or noninflamed controls. Cytokine amounts are expressed as the amounts released by peripheral T cells incubated with CM minus cytokine amounts in CM alone, in pg/mL. For each blood donor, cytokine amounts released by peripheral T cells incubated with CM from CC patients were compared with the amounts released by peripheral T cells incubated with CM from noninflamed controls. Each symbol represents data from one donor and the data points from CC and noninflamed controls for each donor are connected with a line. *  = *P* < 0.05 versus noninflamed controls.

To mimic* in vitro* the exposure of peripheral T lymphocytes that have newly arrived into the colonic mucosa and to determine whether the local intestinal milieu affects T-cell activation and differentiation, we investigated T-cell cytokine production by α-CD3 plus α-CD28 stimulated CD4^+^ peripheral blood T cells in the presence of soluble factors from the intestinal mucosa. There was significantly increased production of the proinflammatory cytokines IFN-*γ*, IL-17A, IL-6, and IL-1*β* in the presence of CM generated by culture of denuded biopsies (DNB-CM) from the colon of collagenous colitis patients, compared to DNB-CM from noninflamed controls (Figure 1). This was evident with the lower protein concentration of CM tested for IL-17A, IL-6, and IL-1*β* and with the higher concentration of CM tested for IFN-*γ* production.

We also noted a significantly increased production of the anti-inflammatory cytokines IL-4 and IL-10 in the presence of DNB-CM from CC patients compared to noninflamed controls, with the lower protein concentration of CM (Figure 1).

In contrast, no significant differences were noted for TGF-*β* production by peripheral CD4^+^ T cells in the presence of DNB-CM from CC patients compared to noninflamed controls (data not shown).

**Figure 2:**
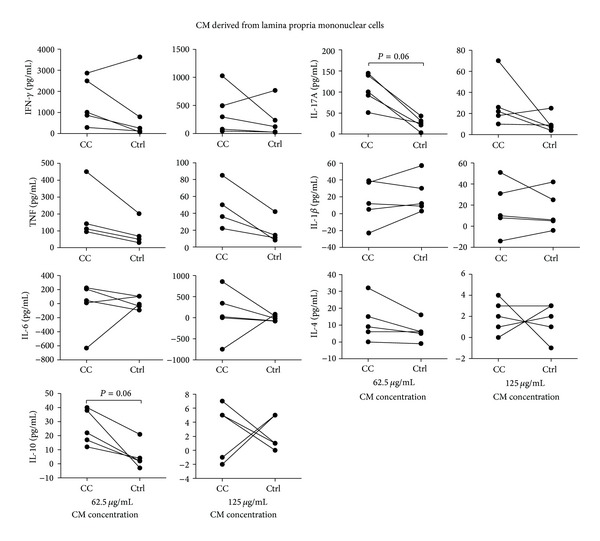
Luminex analysis for IL-1*β*, IL-4, IL-6, IL-10, IL-17A, IFN-*γ*, and TNF production by anti-CD3 plus anti-CD28 stimulated CD4^+^ peripheral blood T cells (*n* = 5; for TNF analysis, *n* = 4) cultured for 3 days in the presence of two concentrations of CM derived from lamina propria mononuclear cells (LPMC) from inflamed CC mucosa or noninflamed controls. Cytokine amounts are expressed as cytokine amounts released by peripheral T cells incubated with CM minus cytokine amounts in CM alone, in pg/mL. For each blood donor, cytokine amounts released by peripheral T cells incubated with CM from CC patients were compared with the amounts released by peripheral T cells incubated with CM from noninflamed controls. Each symbol represents data from one donor and the data points from CC and noninflamed controls for each donor are connected with a line.

In general, LPMC-CM had less impact on peripheral CD4^+^ T-cell activation and differentiation. A trend towards increased production of IL-17A and IL-10 (both *P* = 0.06) was noted in the presence of LPMC-CM from CC patients compared to noninflamed controls in the lower protein concentration (Figure 2). The other cytokines investigated were not significantly altered (Figure 2).

### 3.2. Reduced Inhibition of CD4^+^ Peripheral T-Cell Proliferation in the Presence of Conditioned Media from Colonic Mucosa from CC Patients

**Figure 3:**
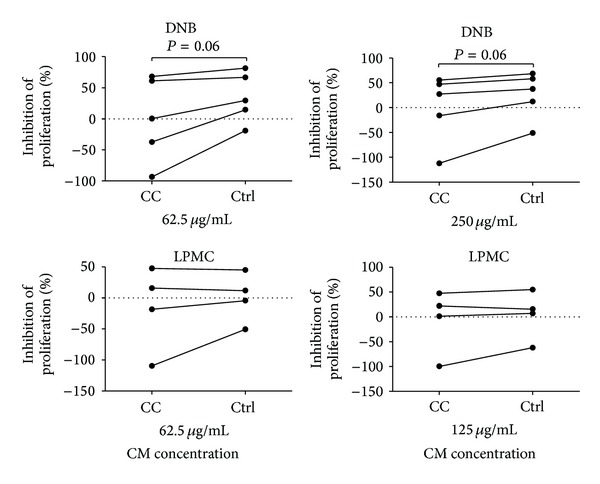
Proliferation of anti-CD3 plus anti-CD28 stimulated CD4^+^ peripheral blood T cells (*n* = 5) cultured in the absence or presence of two concentrations of conditioned medium (CM) derived from denuded biopsies (DNB) or from lamina propria mononuclear cells (LPMC) from inflamed CC mucosa or noninflamed controls, as analysed on day 3 of culture. Data are presented as percentage inhibition of proliferation compared to cells cultured in the absence of CM. Each symbol represents data from one blood donor and the data points from CC and noninflamed controls for each donor are connected with a line. For LPMC fraction, CD4^+^ peripheral blood T cells from four blood donors were tested for proliferation analysis.

We next investigated the ability of soluble factors from the colonic mucosa to inhibit T-cell proliferation, as this has previously been demonstrated* in vitro* [4, 16, 17]. A tendency towards reduced proliferation inhibition of peripheral CD4^+^ T cells was noted in the presence of CM from culture of denuded biopsies (DNB-CM) (*P* = 0.06) from inflamed CC patients compared to noninflamed controls (Figure 3). This was evident with both total protein concentrations of DNB-CM tested for peripheral T-cell proliferation.

In contrast, no differences in proliferation inhibition were observed in the presence of LPMC-CM from noninflamed controls compared to CC patients (Figure 3).

### 3.3. Enhanced IL-1*β* and IL-6 Levels in Conditioned Medium from Inflamed CC Mucosa

As T-cell differentiation and function are regulated by different cytokines, we next analysed eight cytokines in pooled conditioned medium from DNB and LPMC fractions derived from inflamed CC mucosa compared to controls. We found more than twofold and eightfold increased levels of IL-6 and IL-1*β*, respectively, in DNB-CM from CC patients compared to controls, whereas no alterations were found in the levels of IFN-*γ*, IL-17A, TNF, IL-4, and IL-10 (Table 1) or TGF-*β* (data not shown). Similar trends of increased levels of IL-6 and IL-1*β* were noted in LPMC-CM from CC patients compared to controls, though it was investigated in pooled CM from only two CC patients (data not shown).

## 4. Discussion

We here report on a novel* in vitro* model for analysis of the impact of the soluble factors from the colonic mucosa of CC patients on peripheral T lymphocyte activation and differentiation. We found that despite the subtle inflammation in the mucosa of collagenous colitis patients, not visible by the naked eye upon colonoscopy, soluble factors in the mucosa are sufficient to significantly enhance the production of IFN-*γ*, IL-17A, IL-6, IL-1*β*, IL-4, and IL-10 by peripheral CD4^+^ T cells exposed to them* in vitro*. This is the first study to investigate the role of soluble factors from the intestinal mucosa of CC patients in the regulation of T cells, where this novel system reflects the impact of* in vivo* exposure of newly recruited peripheral blood T cells to soluble factors in the colonic milieu of inflamed mucosa from CC patients compared to normal mucosa.

CM from denuded biopsies from inflamed CC mucosa induced increased production of both pro- and anti-inflammatory cytokines by peripheral T cells. As microscopic colitis is subtler compared to ulcerative colitis and Crohn's disease, this may suggest that the colonic microenvironment in CC promotes production of anti-inflammatory cytokines to counterbalance inflammatory responses. Apparently this is not sufficient to keep the inflammation at bay. A study on the effects of stroma conditioned medium from Crohn's patients mucosa on cytokine production by T cells demonstrated increased IFN-*γ* and IL-17 production but provided no data on the effect of anti-inflammatory cytokine production [4].

No differences were noted in TGF-*β* production by peripheral CD4^+^ T cells in the presence of CM from CC patients compared to controls. Whereas TGF-*β* in the normal mucosa likely suppresses T-cell function, the significantly increased amounts of IL-6 and IL-1*β* in the inflamed CC mucosa instead likely promote differentiation of proinflammatory Th17 cells [7, 18] producing large amounts of IL-17A. The colonic milieu from CC patients might also promote differentiation of peripheral CD4^+^ T cells into IL-17/IFN-*γ* double producing Th17/Th1 cells that have been suggested to mediate gut inflammatory processes [19–21], corroborating our findings of enhanced levels of both IL-17A and IFN-*γ*.

We found a trend towards reduced inhibition of T-cell proliferation by soluble factors from denuded biopsies from the colonic mucosa of CC patients compared to controls. This is in accordance with the findings by Huff et al. on stroma conditioned medium derived from inflamed Crohn's mucosa [4]. Older studies have demonstrated that the mucosal microenvironment reduces the proliferative responses of lamina propria lymphocytes to antigen receptor stimulation [17, 22] but they are still active in their helper and cytolytic functions [22, 23]. The present study together with the study by Huff et al. [4] indicates that these effects are at least partly imprinted in the T lymphocytes by the local milieu, rather than an intrinsic characteristic.

In contrast to CM from denuded biopsies, lamina propria mononuclear cell- (LPMC-) CM from CC patients did not affect T-cell proliferation compared to LPMC-CM from controls. These different effects of the two types of CM are unclear and further experiments need to be performed to elucidate the differences in the composition of the CMs.

Despite our observed enhanced production of IL-10 by peripheral T cells in the presence of CM from CC patients, known to inhibit both T-cell proliferation and cytokine production [24], we found neither reduced proliferation of peripheral T cells nor production of proinflammatory cytokines. This indicates that other immunoregulatory molecules drive synthesis of these proinflammatory cytokines in collagenous colitis.

The production of cytokines did not increase with higher total protein concentrations in the CM. One explanation could be the presence of inhibiting and/or toxic factors in the CM limiting the T-cell responses. In addition, various molecules have different optimal concentrations for their function and high concentrations can limit their activity. To further elucidate this and explain the differences observed on CD4^+^ T-cell differentiation we want to investigate a larger panel of cytokines and compare the protein profile between CM from CC patients and that from noninflamed controls and between DNB and LPMC fractions by proteomics.

In conclusion, we have set up an* in vitro* model for analysis of the impact of the soluble factors from the colonic mucosa of CC patients on peripheral T lymphocyte activation and cytokine production. Despite the subtle inflammation in CC, our data demonstrate significant alterations in cytokine production by peripheral CD4^+^ T cells in the presence of mucosa-derived soluble factors from CC patients compared to controls. One of our future goals is to test this* in vitro* model on differentiation of CD8^+^ T cells, as we have previously reported on their increased numbers in the colonic mucosa of CC patients. We also want to evaluate its use in evaluating the effect of drugs, including those in present use, on the colonic mucosal milieu and the lymphocytes there within, thereby facilitating the decision on optimum molecules as well as doses required for suppression of T-cell inflammatory responses.

## Figures and Tables

**Table 1:** Protein levels (pg/mL) of cytokines analysed in pooled condition medium (CM) derived from denuded biopsies (DNB) from inflamed collagenous colitis (CC) mucosa or normal mucosa (control, *n* = 20; CC, *n* = 7).

	IFN-*γ*	IL-17A	TNF	IL-1*β*	IL-6	IL-4	IL-10
CC-DNB-CM	772	40	97	1831	9326	12	511
Ctrl-DNB-CM	509	36	70	228	4138	11	525
